# Impact of Plant Species on the Synthesis and Characterization of Biogenic Silver Nanoparticles: A Comparative Study of *Brassica oleracea*, *Corylus avellana*, and *Camellia sinensis*

**DOI:** 10.3390/nano14231954

**Published:** 2024-12-05

**Authors:** Gülçin Demirel Bayik, Busenur Baykal

**Affiliations:** Department of Environmental Engineering, Faculty of Engineering, Zonguldak Bulent Ecevit University, Zonguldak 67000, Turkey; busenurbaykall@gmail.com

**Keywords:** nanoparticle, green synthesis, silver nanoparticle, plant extract

## Abstract

The choice of plant species is crucial, as different plants provide unique biomolecules that influence nanoparticle characteristics. Biomolecules in plant extracts, such as proteins, amino acids, enzymes, polysaccharides, alkaloids, tannins, phenolics, saponins, terpenoids, and vitamins, act as stabilizing and reducing agents. This study explores the synthesis of silver nanoparticles (AgNPs) using leaf extracts from collard greens (*Brassica oleracea* var. *acephala*), hazelnut (*Corylus avellana* var. *avellana*), and green tea (*Camellia sinensis*). NPs were synthesized using silver nitrate (AgNO_3_) solution at two different molarities (1 mM and 5 mM) and characterized by UV–Vis spectroscopy, XRD, TEM, and FTIR. The Surface Plasmon Resonance (SPR) peaks appeared rapidly for hazelnut and green tea extracts, within 30 and 15 min, respectively, while collard greens extract failed to produce a distinct SPR peak. X-Ray Diffraction confirmed the formation of face-centered cubic silver. TEM analysis revealed high polydispersity and agglomeration in all samples, with particle size generally decreasing at higher AgNO_3_ concentrations. However, hazelnut extract showed a slight increase in size at higher molarity. Among all samples, green tea-derived AgNPs synthesized with 5 mM AgNO_3_ were the smallest and least polydisperse, highlighting the significant role of plant type in optimizing nanoparticle synthesis.

## 1. Introduction

Nanotechnology is the practical application of nanoscience, which involves the synthesis, characterization, and use of materials ranging in size from 1 to 100 nm [1,2]. Nanoparticles, which can be synthesized from various materials such as carbon, ceramics, polymers, and metals, are widely utilized in bioengineering due to their unique surface properties [3,4]. In recent years, metal nanoparticles have gained significant attention in the field of nanotechnology due to their optical, electrical, and catalytic properties [5,6]. These nanoparticles are employed in various technology and industry sectors as nanoadsorbents [7], biosensors [8], catalysts [9], and antibacterial agents [10], etc. Metal nanoparticles come in various forms, such as silver, gold, zinc, cadmium sulfide, copper, iron, and titanium dioxide, each possessing distinctive properties [11]. Silver nanoparticles (AgNPs) have been one of the most popular subjects of study in recent decades due to their broad range of applications in various fields of biomedical science, especially antiviral, antimicrobial, antifungal, antioxidant, and anti-cancer [12,13,14,15,16,17,18], as well as biosensors [19], dental [20,21], electronic applications [22,23], and water treatment [24,25].

Nanoparticles can be synthesized by chemical, physical, and biological approaches. However, nowadays these methods are not preferred by researchers due to harmful effects to the environment, toxicity, high-energy consumption, and cost [2,25,26]. For these reasons, nanoparticle synthesis by the biological method, also called green synthesis, is widely used. This method is an environmentally friendly, cost-effective, safe, and biocompatible approach. Nanoparticle synthesis is realized by combining simple structures by using microorganisms such as fungi, bacteria, algae, and plants in biological synthesis (green synthesis) [25,27,28,29]. The impact of process parameters such as concentration, initial pH, and time has been investigated in order to improve particle stability, increase yield, and stop product aggregation [30,31]. The capacity of metal nanoparticles to stay distributed in liquids without exhibiting agglomeration or coagulation is a crucial characteristic in their manufacture. Hydrophilic surfactants or capping agents, such as proteins and amino acids, are typically used to stabilize particles when creating nano-silver for biological applications [32]. Different microbial species may use different combinations of proteins and enzymes in the synthesis process, which can affect the control, localization, and shape of nanoparticles [33]. It is commonly known that the kind and properties of the proteins involved affect the properties of the produced nanoparticles [34].

Synthesis with microorganisms is not much preferred due to high level of aseptic requirements and preservation. Also, the use of plants provides high-scale nanoparticle synthesis and obtaining nanoparticles of desired shape and size. Biomolecules such as proteins, amino acids, enzymes, polysaccharides, alkaloids, tannins, phenolics, saponins, terpenoids, and vitamins in plant extracts act as stabilizing and reducing agents, reducing metal ions and providing the formation of metal ions [35,36]. Because of the high bioreduction potential of plant biomass or plant extracts, the synthesized nanoparticles can be synthesized more stable and with higher speed. Nanoparticles of silver, gold, and many other metals are synthesized in this method [12,37]. Examples of silver nanoparticles synthesized with different plant leaf extracts are *Ziziphora tenuior* [38], *Camellia sinensis* [39], *Terminalia bellerica* [40], *Aloe vera* [41], and *Cuminum cyminum* [42].

NPs that exhibit greater stability, cost-effectiveness, and a controlled release rate have been effectively utilized for removing inorganic anions [43], heavy metals [44], organic pollutants [45], and bacteria [46] from water, demonstrating significant potential for use in water and wastewater treatment applications. Over the past decade, the employment of nanoparticles for dye removal from wastewater has gained significance due to their large surface area, high adsorption properties, reduced diffusion resistance, and faster attainment of equilibrium [47].

The main objective of this study was to investigate the effect of different plant species, collard (*Brassica oleracea* var. *acephala*), hazelnut (*Corylus avellana* var. *avellana*), and green tea (*Camellia sinensis*), on the production of nanoparticles, comparing their influence on the size, shape, and crystalline properties of the synthesized nanoparticles. The study aimed to compare the synthesis conditions and examine the size, shape, and crystalline properties of the synthesized nanoparticles, highlighting the role of the selected plant extracts. The properties of the synthesized nanoparticles were analyzed using various instrumental techniques, including Transmission Electron Microscopy (TEM), Fourier Transform Infrared Spectroscopy (FTIR), and X-Ray Diffraction (XRD). In this study, silver nanoparticles were synthesized with three different plant extracts, collard (*Brassica oleracea* var. *acephala*), hazelnut (*Corylus avellana* var. *avellana*), and green tea (*Camellia sinensis*), under different conditions. Comparative analyzes were carried out based on synthesis conditions, size, shape, and crystalline properties of synthesized nanoparticles which emphasize the importance of the selected plant extract. The properties of the synthesized nanoparticles were investigated through various instrumental techniques including TEM, FTIR, and XRD.

## 2. Materials and Methods

All the chemicals used were of analytical grade. Folin–Ciocalteu phenol reagent, anhydrous sodium carbonate (Na_2_CO_3_), and anhydrous gallic acid for synthesis were obtained from Merck (EMD Milipore Corporation, Darmstadt, Germany), and Silver nitrate (AgNO_3_) and ethanol (≥99.8% GC Grade) were obtained from Sigma Aldrich Merck (EMD Milipore Corporation, Darmstadt, Germany),. All the spectroscopic measurements were performed by Shimadzu UV-1800 spectrophotometer (Shimadzu Corporation, Kyoto, Japan). A magnetic stirrer (Sci Finetech Co. FTHPM-62, Seoul, Republic of Korea) was used for plant extract preparation and Gerhardt THO 500/1 Thermoshake (Königswinter, Germany) was used for agitation during phenolic content analysis.

### 2.1. Preparation of Plant Extracts

Collard greens (CG) (*Brassica oleracea* var. *acephala*) and hazelnut (HN) (*Corylus avellana* var. *avellana*) leaves were collected from gardens within the Zonguldak province, while commercial green tea (GT) (*Camellia sinensis*) leaves were obtained from a local market. To ensure the removal of any surface contaminants, the leaves were first washed with tap water and then with distilled water. Subsequently, the cleaned leaves were dried under sunlight for several days until the moisture content decreased and the leaves became easily crumbled and passed through a sieve. Then they were cut into small pieces and passed through an 18-mesh sieve to ensure a homogeneous size distribution. The prepared dried leaves were weighed (12 g) and added to 180 mL of distilled water for extraction. The resulting solution underwent a controlled boiling process, facilitated by a magnetic stirrer, at 100 °C for 10–15 min, until a color change from yellow to brown occurred. Post-boiling, the plant extract was allowed to cool to room temperature, and, subsequently, it was subjected to filtration through Whatman No 1 filter paper with a pore diameter of 11 μm, thereby rendering it amenable for nanoparticle synthesis. The same procedure was applied for each plant sample.

### 2.2. Total Phenolic Content (TPC) of Plants

The determination of the total phenolic content (TPC) of the plants, which influences the synthesis of nanoparticle formation, was conducted utilizing the Folin–Ciocalteu method. In the extraction process, 1 g of plant leaves was precisely weighed and introduced into ethanol solutions with concentrations of 30%, 40%, and 60%. The resulting mixture underwent agitation at 100 rpm for 30 min using a Thermoshake and was subsequently filtered through Whatman No:1 filter paper.

Following filtration, 1.5 mL of Folin–Ciocalteu solution (10% in water) was added to 200 μL of the diluted sample (diluted 25-fold with solvent) and allowed to stand at room temperature for 5 min. Subsequently 1.5 mL of anhydrous sodium carbonate Na_2_CO_3_ (75 g/L in water) was added and rapidly mixed, followed by incubation in the dark at room temperature for 1 h. The formation of a blue color in the sample indicated the presence of phenolic compounds. Absorbance values at 725 nm were measured using a spectrophotometer against a calibration curve prepared using 0–100 mg/L gallic acid solutions. The same procedures were repeated for the gallic acid solutions. Distilled water was used as a control sample. The obtained results were expressed as milligrams of gallic acid equivalents per gram of dry weight (mg GAE/g dw).

### 2.3. Nanoparticle Synthesis and Characterization

The nanoparticle synthesis process involved the reaction of plant extracts, diluted with distilled water at varying ratios (no dilution, 1:1, 1:4, 1:10), with silver nitrate solutions prepared at two distinct molarities (1 mM and 5 mM). The addition of AgNO_3_ solution to the extract occurred at a controlled-extract-to-AgNO_3_ ratio of 1:10. The appearance of a noticeable color change from yellow to dark brown within the initial 15–20 min signified the successful formation of silver nanoparticles (AgNPs). Nanoparticle synthesis was monitored by taking samples at regular intervals over a 24 h duration. Their absorbances were measured using a Shimadzu UV-1800 spectrophotometer. Distilled water served as the control sample throughout the experimentation.

To ensure comprehensive and detailed analysis of structural and morphological attributes of the synthesized nanoparticles, produced nanoparticles were investigated through various analytical techniques, including Transmission Electron Microscopy (TEM), X-Ray Diffraction Spectrometer (XRD), and Fourier Transform Infrared Spectroscopy (FTIR). These analyses were carried out in the Middle East Technical University (METU) Central Laboratory. Interventionary studies involving animals or humans, and other studies that require ethical approval, must list the authority that provided approval and the corresponding ethical approval code.

## 3. Results and Discussion

### 3.1. UV–Vis Spectra of NPs

The synthesized silver nanoparticles from three different plant species were named as CG-AgNPs (collard greens silver nanoparticles), HN-AgNPs (hazel nut silver nanoparticles) and GT-AgNPs (green tea silver nanoparticles) and their formations were confirmed by the UV–Vis spectra, which showed absorption peak between 450 and 460 nm (Table 1). The total phenolic content of the CG, HN, and GT were calculated as 27.4, 53.9, and 89.4 mg GAE/g, respectively.

**Figure 1:**
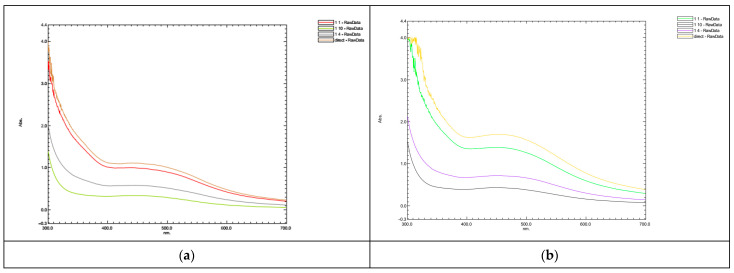

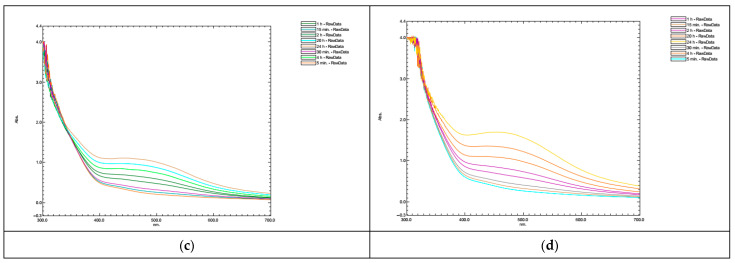
Absorption spectra of nanoparticles synthesized by collard greens extract (**a**,**c**) 1 mM AgNO_3_; (**b**,**d**) 5 mM AgNO_3_.

**Figure 2:**
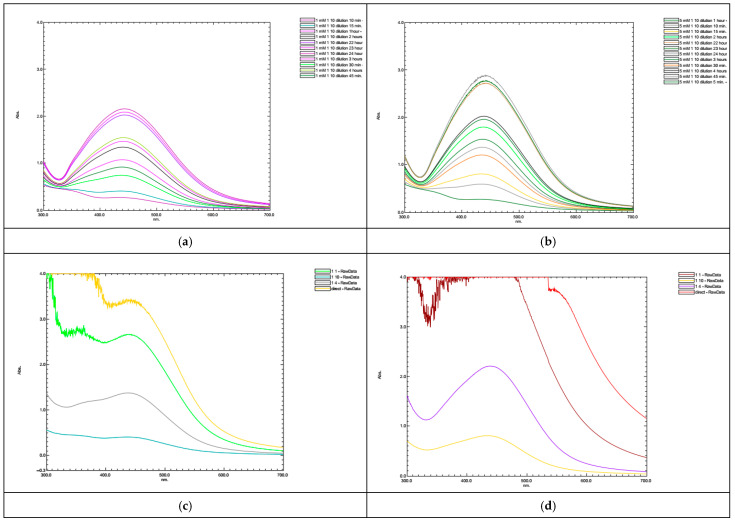
Absorption spectra of nanoparticles synthesized by hazelnut extract (**a**,**c**) 1 mM AgNO_3_; (**b**,**d**) 5 mM AgNO_3_.

**Figure 3:**
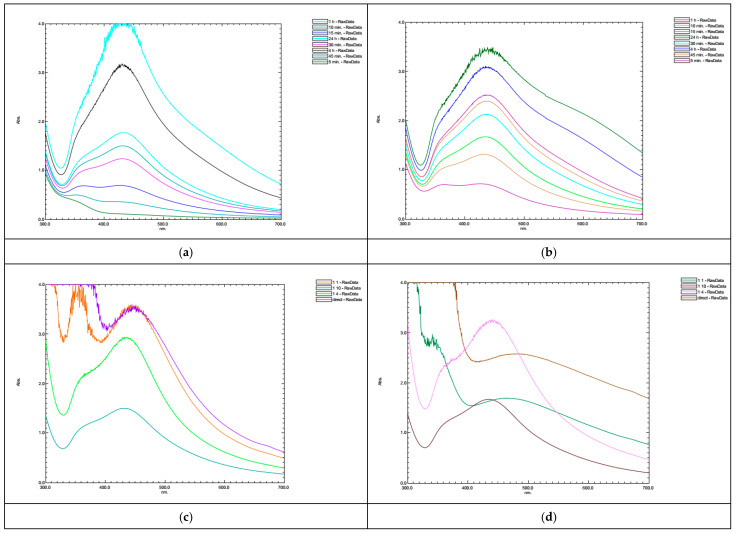
Absorption spectra of nanoparticles synthesized by greentea extract (**a**,**c**) 1 mM AgNO_3_; (**b**,**d**) 5 mM AgNO_3_.

The formation of nanoparticles for varying AgNO_3_ concentrations and diverse dilution ratios are presented in Figure 1, Figure 2 and Figure 3. The Surface Plasmon Resonance bands are influenced by several factors including the shape, morphology, size, dielectric environment, and composition of the synthesized nanoparticles (NPs) [48]. The broadening of the absorption band provides information about the shape and size homogeneity of the nanostructures in the sample. Wider absorbance bands represent the plural distribution of nanoparticle size, and narrower absorbance bands represent a single distribution [49,50]. Also, when there is a decrease in the size of nanoparticles, the SPR band tends to shift towards the blue-shift (short wavelength, 400–500 nm) region, while as the particles’ diameter increases, there is a tendency for a red-shift (long wavelength, 600–700 nm) of the SPR band [50,51].

In the current study, the peaks observed under various experimental conditions predominantly exhibited single Surface Plasmon Resonance (SPR) bands, suggesting the predominance of spherical-shaped nanoparticles in the synthesized samples although widening or broadening of the spectra were observed.

#### 3.1.1. Collard Greens Silver Nanoparticles (CG-AgNPs)

The analysis of the Surface Plasmon Resonance (SPR) band for CG-AgNPs is presented in Figure 1a–d. Specifically, the results for 1 mM and 5 mM AgNO_3_ concentrations are depicted in Figure 1a,c and b,d, respectively. For the 1 mM AgNO_3_ solution (undiluted), the SPR band initiation is observed at 450 nm approximately 240 min after the starting of the reaction. Conversely, in the case of the 5 mM AgNO_3_ solution (undiluted), a similar appearance of the SPR band is noted within the same timeframe, albeit slightly shifted to 455 nm. These observations are illustrated in Figure 1a,b correspondingly. However, it is noteworthy that a distinct and narrow peak characteristic of conventional SPR bands was not achieved for nanoparticles synthesized from collard greens. The mechanism underlying the synthesis involves the bioreduction of silver ions facilitated by the presence of a reducing agent, which transforms Ag+ ions from the AgNO_3_ solution into nanoscale Ag^0^ [52].

In this study, it was observed that the scarcity of phenolic groups within collard green leaves may act as a limiting factor in the production of nanoparticles.

#### 3.1.2. Hazel Nut Silver Nanoparticles (HN-AgNPs)

The evaluation of absorption spectra for HN-AgNPs is given in Figure 2a–d. The emergence of the initial Surface Plasmon Resonance (SPR) peak for the 1 mM AgNO_3_ concentration was noticeable just after 30 min, with a gradual increase in peak intensity observed within 4 h. The figure reveals that NP production is completed by approximately the 22nd hour. For the 5 mM concentration, peak formation is observed quite quickly, occurring within the initial 10–15 min. Similar to the lower concentration, its intensity amplifies over time, reaching completion by the 22nd hour.

The influence of the dilution ratio is presented in Figure 2c,d. At lower dilution ratios, a discernible and uniform UV absorbance peak could not be obtained, attributed to the abundance of reducing agents in the environment with limited silver ions available in return. This imbalance signifies that while there are abundant reducing agents capable of converting silver ions to Ag^0^, there exists an inadequacy of silver ions for this transformation process. Therefore, the surplus of reducing agents disrupts the equilibrium of the reaction, impeding the formation of a stable UV absorbance peak. Consequently, achieving homogeneous nanoparticle production becomes challenging at lower dilution ratios, and the establishment of a distinct UV absorbance peak becomes unattainable [53].

For both molarities, the attainment of a uniform absorbance peak was confined to a dilution ratio of 1:4. Nevertheless, it was observed that peak formation commenced even at higher dilutions, particularly when dealing with higher concentrations of silver nitrate.

#### 3.1.3. Green Tea Silver Nanoparticles (GT-AgNPs)

Among the three plant species, green tea exhibits the highest phenolic content, which correlates with the swift formation of NPs absorbance peaks, as illustrated in Figure 3. At a silver nitrate concentration of 1 mM, a broad absorbance peak emerges as early as the 15th minute, indicating the presence of AgNPs with broader size distributions [53]. After the 15th minute, a wide broad band occurred attributed to plural dispersion of synthesized NPs. Over the 1 h, there is a slight increase in intensity, but no significant narrowing of the peak is observed. It is only after approximately 4 h that narrower peaks become observable. At a concentration of 5 mM AgNO_3_, the formation of a broad absorption spectrum is noticeable shortly after the initial 5 min with plural dispersion. Similarly, as the first hour elapses, the intensity of the peaks increases and they become narrower. However, achieving smooth absorption peaks at the 4th and 24th hours proves unattainable. Nanoparticle formation peaks are evident at plant dilution ratios of 1:4 and 1:10 for both concentrations of silver nitrate. Remarkably, these peaks appear more distinct, particularly at the 5 mM concentration.

The faster completion of the reaction for hazelnut and green tea plants is associated with the high reduction potentials of the active components of the plant extracts, as evidenced by the calculated phenolic content.

### 3.2. XRD Analysis of NPs

**Figure 4:**
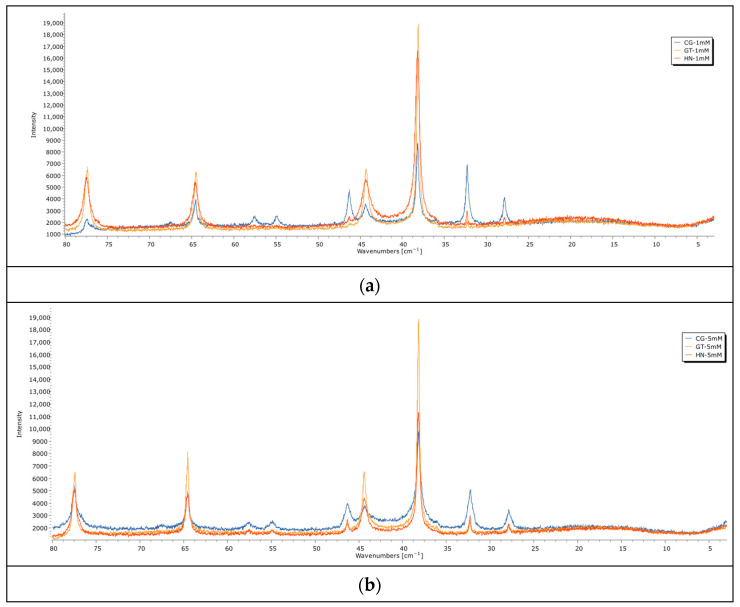
XRD patterns of produced nanoparticles (**a**) 1 mM three plant species; (**b**) 5 mM three plant species.

**Figure 5:**
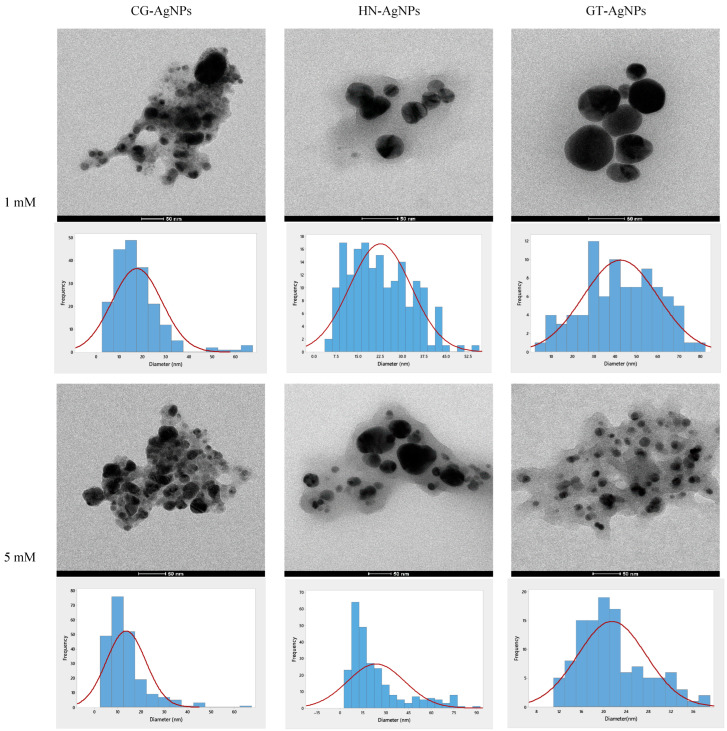
TEM, particle size distribution, and EDX spectrum of nanoparticles.

The investigation into the crystalline structure and phase purity of the synthesized silver nanoparticles (AgNPs) was carried out through X-Ray Diffraction (XRD) analysis spanning the 2θ range of 3–80 degrees. The XRD pattern, delineating the AgNPs synthesized via the reaction between a silver salt solution and plant extracts, and comparing the molarity effect as well as differences among plant species, is depicted in Figure 4a,b. The XRD plot of the AgNPs separately for 1 mM and 5 mM are given in Supplementary Materials Figure S1. In X-Ray Diffraction (XRD) analysis, the intensity of peaks serves as an indicator of the atomic density within specific crystal planes. Consequently, the synthesized silver nanoparticles (AgNPs) utilizing plant extracts exhibited prominent Ag peaks around 38°, 45°, 64°, and 77°, corresponding to the crystal planes (1,1,1), (2,0,0), (2,2,0), and (3,1,1) of the face-centered cubic silver structure (ICDD 893722) [54]. AgCl nanoparticle peaks were also observed to coexist with Ag nanoparticles, giving the typical peaks at around 27.8°, 32.2 °, 46.2°, 54.95°, and 57.51° corresponding to the crystal planes (1,1,1), (2,0,0), (2,2,0), and (3,1,1) (ICDD 311238). XRD results clearly show that the AgNPs prepared by the different plant extracts are observed to be well crystallized due to the sharpness in the major Ag peak (1 1 1). Figure 4 gives the observed nanoparticle phases prepared by using different plant types. A remarkable observation in the X-Ray Diffraction (XRD) results is the significantly high intensity of the AgCl peaks at 27.8 and 32.2 degrees in the CG-AgNPs. Furthermore, the peak at 32.2 is quite close to 38.1 degrees, corresponding to Ag, and its intensity is higher than other peaks in the spectrum (especially at a concentration of 1 mM silver nitrate). The results are in accordance with EDAX (Figure 5) results that give both silver and chlorine. In the HN-AgNPs, the peaks at 27.8 and 32.2 degrees for the 5 mM silver nitrate concentration are more prominent compared to those for the 1 mM. However, they are considerably lower in intensity compared to the peak at 38 degrees. Another significant observation is that silver nanoparticles produced with 1 mM silver nitrate and green tea extract do not contain AgCl and consist solely of pure Ag components. However, in the case of a 5 mM silver nitrate concentration, two very small peaks were observed at 32.2 and 46.2 degrees. These findings underscore the influence of the choice of plant extract on the crystalline structure and peak intensities of the synthesized AgNPs. Notably, hazelnut leaves demonstrate a remarkable enhancement compared to green tea and collard greens, suggesting potential variations in the chemical composition or inherent properties of the plant extracts contributing to these differences in nanoparticle synthesis.

#### 3.2.1. Transmission Electron Microscopy (TEM) and Size Distribution

Transmission electron microscopy (TEM) images and particle size distributions of the nanoparticles are presented in Figure 5. Size distribution analysis was performed using ImageJ software version 1.51. The TEM analysis revealed that all synthesized nanoparticles exhibited high polydispersity and were quite agglomerated. It was observed that the particle size decreased with increasing silver nitrate molarity. However, an exception was noted with hazelnut leaf extract, where a slight increase in nanoparticle size was observed with higher concentrations. The most pronounced size variation in response to silver nitrate molarity was observed in nanoparticles synthesized with green tea extract. At 1 mM concentration, the average nanoparticle size was 42 ± 17.46 nm, whereas at 5 mM concentration, the size decreased significantly to 12.7 ± 11.11 nm. Such a dramatic size reduction was not observed in the other two plant species with varying silver nitrate concentrations. At 1 mM Ag(NO_3_), the majority of the particles were spherical; however, there were both very large and very small particles present. At 5 mM, nanoparticles exhibited a range of sizes, with more irregular shapes compared to the 1 mM concentration. Rod-like, triangular, and oval-shaped nanoparticles were observed. The size distribution was similar across the two different molarities. The HN-AgNPs synthesized using 1 mM Ag(NO_3_) were less aggregated, allowing better observation of the particles. Most nanoparticles were spherical, and the size distribution was more uniform compared to 5 mM. Using 5 mM Ag(NO_3_) solution led to particles with more irregular shapes, with a mixture of very large and very small particles. Predominantly larger GT-AgNPs were observed, with a more uniform size distribution for 1 mM Ag(NO_3_) concentration. There was less agglomeration, and the particles were mostly spherical, with a few rod-shaped nanoparticles. GT-AgNPs synthesized using 5 mM Ag(NO_3_) featured the smallest and least polydisperse nanoparticles among all samples. The majority of the nanoparticles were spherical with reduced agglomeration. Increased reactant molarity resulted in a narrower and more uniform size distribution of nanoparticles. Therefore, it can be concluded that high the capacity of polyphenols may contribute to the functionalization of NPs, which would prevent aggregation [55].

These results differ from findings in the existing literature. For instance, Li et al. [56] reported that increasing reactant molarity led to more variable gold nanoparticle sizes. They also noted that size control could be achieved through pH regulation [56]. Aritonang et al. synthesized silver nanoparticles using extracts of two medicinal plants and reported that the particle size was increased with increased Ag(NO_3_) concentration and the nanoparticles were quite polydispersed. At 1 mM AgNO_3_ concentration, the size of the nanoparticles was 12 ± 2 and 3.2 ± 1.2 for Impatiens balsamina and Lantana camara, respectively. When the concentration was increased to 5 mM, the particle sizes were increased to 20 ± 3.3 and 12 ± 2.1 [57]. Onitsuka et al. synthesized Ag nanoparticles using green tea extract with a particle size of 18.9 ± 4.4 nm [58].

The elemental composition given in the particle size distribution graphs in Figure 5 reveals a strong signal for silver. In addition, carbon and oxygen signals could also be detected which are attributed to polyphenols or any other phytochemical compound containing C and O elements [59].

The Scherrer equation is commonly used to calculate the crystalline size of the nanoparticles [60].

D = kƛ/βcos θ

The calculated crystalline size and particle size of the nanoparticles are given in Table 2.

Although the literature generally reports an increase in particle size with higher silver nitrate molarity, controlling the size and shape of nanoparticles remains challenging. In this study, the particle size and the crystallite sizes were observed to be inversely proportional to the molarity of silver nitrate. This deviation of the particle size from the literature is attributed to the aggregation properties of nanoparticles. Zare’s research on the aggregation and agglomeration of particle nanocomposites demonstrated that an increasing volume fraction typically results in smaller aggregate sizes. However, these smaller aggregates are prone to higher re-agglomeration effects. This suggests that smaller nanoparticles may exhibit greater tendencies for aggregation and agglomeration compared to larger particles [61].

#### 3.2.2. Fourier Transform Infrared Spectroscopy (FTIR)

**Figure 6:**
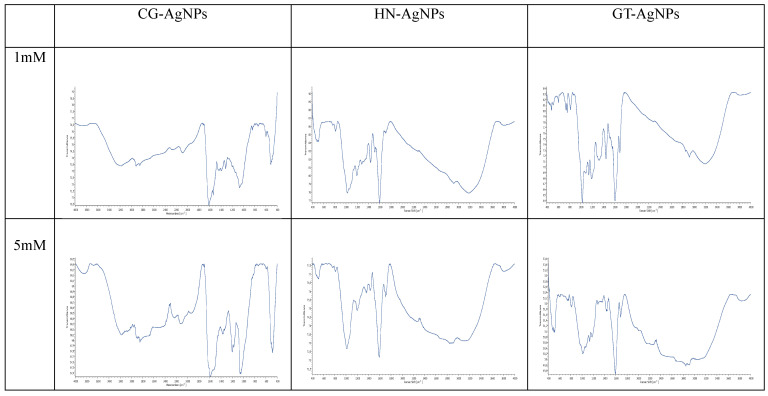
FTIR spectra of produced AgNPs.

Figure 6 represents the FTIR spectrum of AgNPs. The FTIR spectrum of silver nanoparticles displayed three prominent peaks in the regions of 480–510 cm^−1^, 1000–1100 cm^−1^, and 1500–1600 cm^−1^ for all samples. Notably, the band observed between 1500 and 1650 cm^−1^ is due to C=O or C=C stretching of carbonyl groups, suggesting the oxidation of flavonoid/polyphenol compounds [11,37]. The carbonyl groups of amino acid residues and peptides exhibit a strong affinity for binding to silver. Additionally, proteins can bind to nanoparticles either through free amine or cysteine groups. The proteins present on the surface of silver nanoparticles act as capping agents [16].

The vibrational frequencies in the range of 900–1370 cm^−1^ due to C-O-C stretching are associated with various biomolecules present in the leaf extract, such as high phenolic content, primary amines, caffeic acid, and ferulic acid. These biomolecules function as both reducing and stabilizing agents during the biosynthesis of metallic nanoparticles [50]. The observed absorption bands in the regions between 400 and 500 cm^−1^ may correspond to the vibrations of triple bonds in alkynes and C-Cl bonds in alkyl halides [44]. Other peaks between 2700 and 2900 cm^−1^, and 1200 and 1500 cm^−1^, may arise from C-H stretching of heteroaromatic groups or aliphatic groups, mainly lipid and protein.

The results obtained from the FTIR analysis were consistent with those from the UV–vis XRD and EDAX analysis, clearly indicating the presence of polyphenols, such as flavonoids. These compounds play a crucial role in the reduction and stabilization of silver nanoparticles (AgNPs) during their synthesis.

## 4. Conclusions

This study presents a green, cost-effective method for synthesizing silver nanoparticles (AgNPs) using plant extracts from collard greens, hazelnut, and green tea under varying conditions. The synthesized nanoparticles were characterized using UV–Vis, TEM, XRD, and FTIR analyses. Green tea extract demonstrated the fastest nanoparticle formation due to its high phenolic content, while collard greens showed limited reduction efficiency. The XRD and TEM analyses revealed that the synthesized nanoparticles had a face-centered cubic silver structure, with size and polydispersity varying based on plant extract and silver nitrate concentration. The study underscores the importance of polyphenol content in stabilizing nanoparticles and provides useful insights for optimizing green synthesis methods. While many studies have explored AgNP synthesis with plant extracts, this work highlights the specific effects of different plant species and the role of dilution in controlling nanoparticle size and stability.

In the present study, we have used a practical, green, and cost-effective method for the synthesis of silver nanoparticles. The silver nanoparticles were synthesized by using plant leaf extract of collard greens, hazelnut, and green tea under different concentration of extract and AgNO_3_. The obtained nanoparticles were characterized using UV–Vis, TEM, XRD, and FTIR analysis. The SPR bands occurred around 450 nm and 455 nm, for AgNPs. Distinct and narrow SPR peaks typical of conventional nanoparticles were not achieved with collard greens. The bioreduction of silver ions (Ag+) to nanoscale silver (Ag0) is influenced by the limited presence of phenolic groups in collard green leaves, which may hinder effective nanoparticle synthesis. The absorption spectra analysis for HN-AgNPs indicates that the SPR peak reaches completion by the 22nd hour. Lower dilution ratios result in inadequate and uneven UV absorbance peaks due to an excess of reducing agents and insufficient silver ions, disrupting the formation of stable nanoparticles. Consistent absorbance peaks were achieved at a dilution ratio of 1:4, highlighting the importance of balancing silver ion concentration with reducing agents for optimal nanoparticle synthesis. Among the three plant species, green tea demonstrates the fastest nanoparticle formation due to its high phenolic content. X-Ray Diffraction (XRD) analysis of AgNPs synthesized from different plant extracts revealed prominent Ag peaks at 38°, 45°, 64°, and 77°, indicating a face-centered cubic silver structure. AgCl peaks were also observed, particularly in CG-AgNPs. TEM analysis showed high polydispersity and significant agglomeration in the synthesized nanoparticles, with particle size generally decreasing with increasing silver nitrate molarity. An exception was noted with hazelnut leaf extract, where particle size slightly increased at higher concentrations. Green tea extract yielded the smallest and least polydisperse nanoparticles at 5 mM, indicating that high polyphenol content may enhance nanoparticle functionalization and prevent aggregation. The FTIR spectrum of AgNPs revealed prominent peaks at 480–510 cm^−1^, 1000–1100 cm^−1^, and 1500–1600 cm^−1^, indicating the presence of C=O or C=C stretching of carbonyl groups, suggesting oxidation of flavonoid/polyphenol compounds. The findings highlight the vital role of polyphenol content in nanoparticle formation and stability, with green tea extract exhibiting superior performance due to its high phenolic content. The study successfully demonstrates the potential of plant-based methods for producing AgNPs, offering insights into the mechanisms and optimizing conditions for green synthesis. Overall, it contributes valuable knowledge to the field of eco-friendly nanoparticle production, showcasing the varying efficacy of different plant extracts in nanoparticle synthesis and stabilization.

## Figures and Tables

**Table 1:** Maximum SPR values measured for different dilution ratios.

Dilution Ratio	Maximum SPR (nm)					
	CGNPs		HNNPs		GTNPs	
	1 mM	5 mM	1 mM	5 mM	1 mM	5 mM
Direkt	450	455	450	X	450	460
1:1	435	450	445	455	445	455
1:4	445	450	440	440	430	440
1:10	440	450	440	440	430	440

**Table 2:** Maximum SPR values measured for different dilution ratios. Crystalline and particle size of synthesized nanoparticles.

Sample	Molarity	Crystallite Size (nm)	Particle Size (nm)
CG	1 mM	20.71	17.61
	5 mM	18.69	13.61
HN	1 mM	12.39	22.71
	5 mM	20.10	23.60
GT	1 mM	21.54	42.67
	5 mM	29.64	21.45

## Data Availability

Data available on request from the authors.

## Supplementary Materials

The following supporting information can be downloaded at https://www.mdpi.com/article/10.3390/nano14231954/s1, Figure S1: XRD patterns of produced nanoparticles 1 mM vs. 5 mM (a) CG-AgNPs, (b) HN-AgNPs (c) GT-AgNPs.
