# Supplementary material for: Impact of Plant Species on the Synthesis and Characterization of Biogenic Silver Nanoparticles: A Comparative Study of Brassica oleracea, Corylus avellana, and Camellia sinensis
Source: Nanomaterials (Basel). 2024 Dec 5;14(23):1954. doi: 10.3390/nano14231954 (PMC11643465; doi:10.3390/nano14231954)
Supplement: Supplementary file 1 [file nanomaterials-14-01954-s001.zip › nanomaterials-3328500-supplementary.pdf]

SUPPLEMENTARY MATERIAL

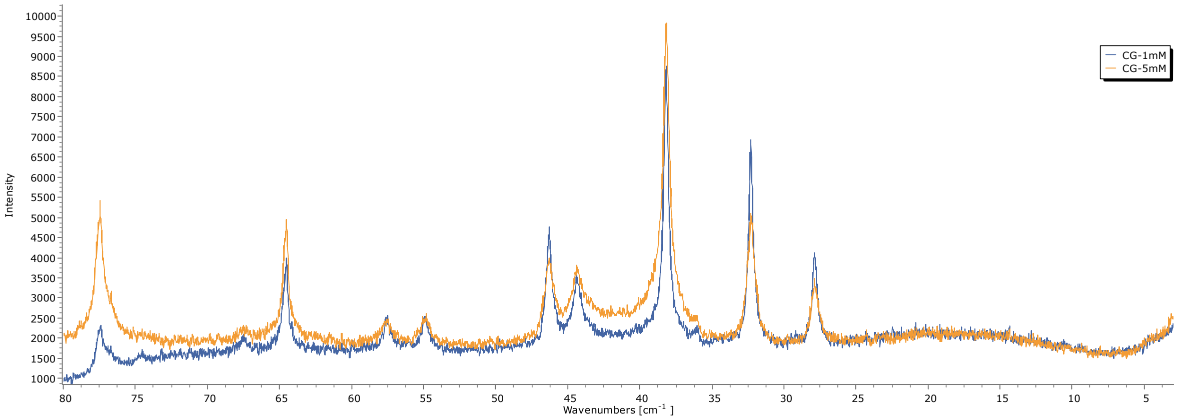

a

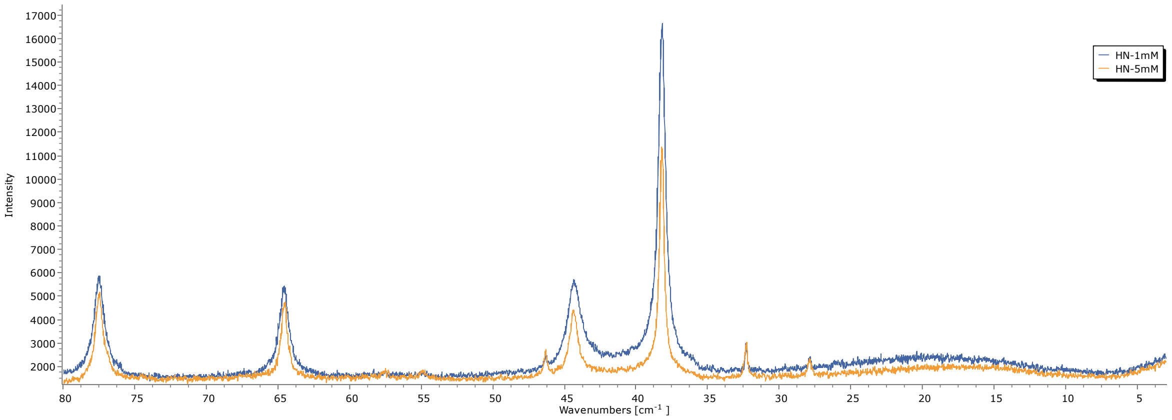

b

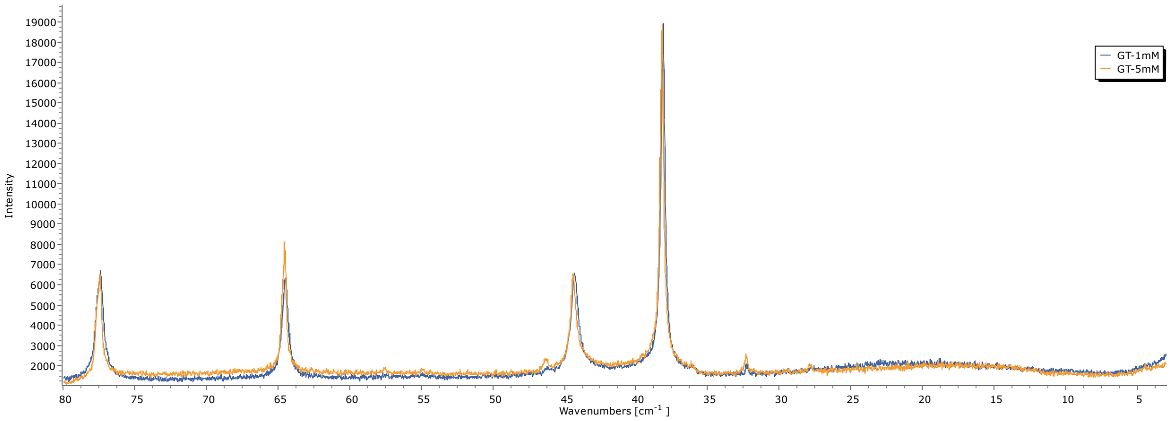

c

Figure S1. 4 XRD patterns of produced nanoparticles 1mM vs 5mM a) CG-AgNPs, b) HN-AgNPs c) GT-AgNPs.
